# Retrieval Analysis of Modern Knee Tumor Megaendoprosthesis Shows Considerable Volumetric Metal Wear Generated at the Rotating Hinge

**DOI:** 10.3390/ma13071519

**Published:** 2020-03-26

**Authors:** Therese Bormann, Sebastian Jäger, J. Philippe Kretzer, Laura Nebel, Lucas Clarius, Georg Omlor, Rudi Bitsch, Burkhard Lehner

**Affiliations:** 1 Laboratory of Biomechanics and Implant Research, Clinic for Orthopedics and Trauma Surgery, Heidelberg University Hospital, 69118 Heidelberg, Germany; Sebastian.Jaeger@med.uni-heidelberg.de (S.J.); philippe.kretzer@med.uni-heidelberg.de (J.P.K.); lauranebel-besigheim@web.de (L.N.); lucas-clarius@t-online.de (L.C.); 2 Clinic for Orthopedics and Trauma Surgery, Heidelberg University Hospital, 69118 Heidelberg, Germany; georg.omlor@med.uni-heidelberg.de (G.O.); burkhard.lehner@med.uni-heidelberg.de (B.L.); 3 ATOS Clinic Heidelberg, 69115 Heidelberg, Germany; rudi.bitsch@atos.de

**Keywords:** metal wear, retrieval study, metal-on-metal articulation, volumetric wear, megaendoprosthesis, total knee arthroplasty, bone tumor

## Abstract

Frequently occurring damage, as well as elevated blood metal ion levels, are reported in relation to a tumor and revision system for total knee arthroplasty (TKA), which applies a rotating hinge coupling with a metal-on-metal (MoM) articulation. As the patient collective for this specific system is small, there is no data on wear generated from the couplings. In this study, wear volume and influencing parameters were investigated at 44 retrieved TKAs with MoM couplings. A scoring system rating frequently occurring abrasive wear between 0 (no wear) and 3 (distinct wear) was established. The wear score was correlated to time in vivo, bone resection length, patient weight and polyethylene inlay damage. Volumetric wear was estimated applying coordinate measurements. An elevated wear score of two or higher was found in 43% of cases. The mean wear rate accounted to 7.8 mm^3^/year. The main influencing coefficient for the extent of wear is time in vivo. We found a tendency for higher wear scores with higher inlay degradation scores. Patient weight and bone resection length did not impact coupling wear. Assessment of wear damage by a semi-quantitative scoring system has proven to be a reliable option for non-destructive coupling evaluation. The generated wear volume is high.

## 1. Introduction

Tumors in the distal femur or the proximal tibia often result in the resection of large bone segments. To salvage the limb, the bone and knee joint are usually reconstructed by endoprosthesis systems [1,2]. Modern systems are constructed modularly in order to address different resection lengths and to permit intra-operational flexibility [1]. As together with the bone also soft tissue and ligaments of the leg are removed, a higher degree of constrain in the knee joint is necessary to ensure stability of the joint. For that, tumor and revisions systems usually couple the distal femur and proximal tibia with a hinge or a rotating hinge system. Generally, the outcome of a fully stabilized and hinged prosthesis is worse than that of a less stabilized knee endoprosthesis [3,4]. Revision rates after five years rise from 3.5% for minimally stabilized total knee arthroplasty (TKA) to 8.1% for the coupled systems, with infection being the most common cause [5].

Aside from infections, mechanical complications like aseptic loosening, structural failure and instability are common reasons for a revision [2,6]. The high incidence of mechanical complications is associated with the coupling, as substantial stresses are transmitted via the coupling mechanism [6,7] and—for the hinged systems—these are further transmitted to the implant–bone interface.

As bone tumors often occur between the second and forth decade of life, the patient collective is considerably younger than that of general primary total knee replacement, which has a mean age of 69 years [5]. It has been shown that the probability for a second complication increases after a first complication requiring revision surgery [8]. Bone tumor patients are therefore very likely to have multiple revision surgeries during the course of their life. Not seldom, the affected limb is sacrificed at some point in the patient’s life [8,9].

The modular universal tumor and revision system (MUTARS^®^, Implantcast, Buxtehude, Germany) is a commonly used endoprosthesis system for tumor patients with good functional clinical results [7,9,10]. The femoral and tibial components of this system are joined by a rotating hinge coupling mechanism, sometimes also referred to as bushing. Despite the satisfactory clinical results of this system, structural complications are reported for up to 30% of the patients [7,9,10,11]. This failure mode includes periprosthetic fracture, breakage of a prosthesis part and wear of the bushing, all requiring a revision operation. The bushing design was changed several times since the introduction of the system in 1995. The latest design uses a metal-on-metal (MoM) coupling mechanism made of cobalt–chromium–molybdenum-alloy. Nevertheless, problems occurring with respect to the coupling mechanism do not seem to be overcome. Bushing wear has still been reported, as well as elevated blood cobalt and chromium levels related to the MoM coupling [9,12]. Even though exchange of the bushing itself is a standard procedure not requiring other parts of the system to be replaced, it affects the patient’s life as an additional surgery. Furthermore, the generated metal particles and ions are released into the human body, where they can cause adverse biological reactions that, in turn, can lead to clinical problems such as soft-tissue necrosis, pseudotumors and particle-induced implant loosening [13,14,15].

In our in-house retrieval registry, we regularly observed (obvious) coupling failures like bushing fracture. Apart from that we noticed marks of abrasive wear on retrieved MUTARS^®^ total knee replacements. As there is no data on wear generated at coupling mechanisms in constrained total knee arthroplasty, we aimed to answer the following research questions in this retrospective study: (1) Is the degree of wear at MoM couplings correlated to implant or patient specific factors? (2) How much wear is actually generated, and can it be quantified?

## 2. Materials and Methods

### 2.1. Retrievals

**Figure 1:**
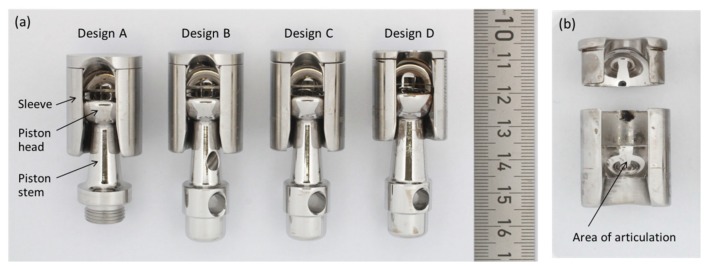
(**a**) Metal-on-metal (MoM) coupling mechanisms consisting of piston and sleeve. The four design variations from our cohort are displayed. Design A refers to the oldest modification, while design D is the most recent. (**b**) The sleeve after separation of the lid for quantitative wear analysis. The arrow indicates the articulating counter face, which was investigated by a coordinate measuring machine (CMM).

We analyzed 44 retrieved MUTARS^®^ prostheses (Implantcast, Buxtehude, Germany) with a metal-on-metal coupling. Retrievals are from our in-house retrieval registry, which is approved by an ethical committee. Implantation of the 44 prostheses was carried out between 1997 and 2018, with explantation between 2001 and 2018. The mean time in vivo was 31.0 ± 38.7 months (median 17.6 months, range 0.4–172.1 months). The coupling is made of cobalt–chromium–molybdenum-alloy (CoCrMo, as specified in DIN ISO 5832-4 [16]), and consists of a sleeve, inside which a piston with a hemispherical head articulates. Among the retrieved couplings we identified four different designs, as displayed in Figure 1a. In the oldest (design A), the piston is fixed within the tibia plateau by a thread. All following designs connect the piston by a stuck bolt to the tibia plateau. Design B is characterized by a bore in the piston stem. Designs C and D are characterized by a bore on top of the hemispherical piston head instead of the one in the stem. Designs C and D further differ in the dimensions of sleeve and piston. In the latest design from our cohort, design D, the piston stem and head are thicker, and the sleeve is shorter as for the designs A, B and C.

### 2.2. Semi-Quantitative Wear Analysis by Scoring System

Failure modes of these prostheses were classified by the Henderson classification. As wear of the coupling occurs independently from the actual failure mechanism of the device, and appears to the naked eye in almost all retrieved samples, we developed a semi-quantitative scoring system to rate coupling wear. The scoring system is based on the Hood and Goldberg scoring systems, which both are well established visual methods to assess damage of retrieved implant parts [17,18]. Arnholt et al. applied a similar scoring system to assess damage of CoCr femoral condyles [19]. As both parts are prone to wear, we rated them individually. Rating was accomplished on a scale between 0 (no wear) and 3 (distinct wear), and the criteria for the point assignment and examples are displayed in Table 1. For the cylindrical sleeve, the external surface was divided into four quadrants, which were rated individually and subsequently averaged. The piston was divided in two zones, the hemispherical head and the stem. Both zones were rated individually from 0 to 3, and subsequently averaged. The score for the whole coupling was determined by averaging the sleeve and piston scores. Scoring was carried out by two independent investigators in order to verify the assessment of wear by the developed scoring scale. All data presented refer to the evaluation of investigator 1.

Couplings were ultrasonically cleaned in two steps prior to score determination. In the first step, cleaning was carried out at 60 °C in a detergent-containing solution for one hour. Subsequently, couplings were immersed in Ethanol for 10 min.

Pre-operative radiographs for metering the bone resection lengths of both the tibia and femur, respectively, were available from 35 patients. In case of the resection of the distal femur and proximal tibia, both values were totalized for further analysis.

**Figure 2:**
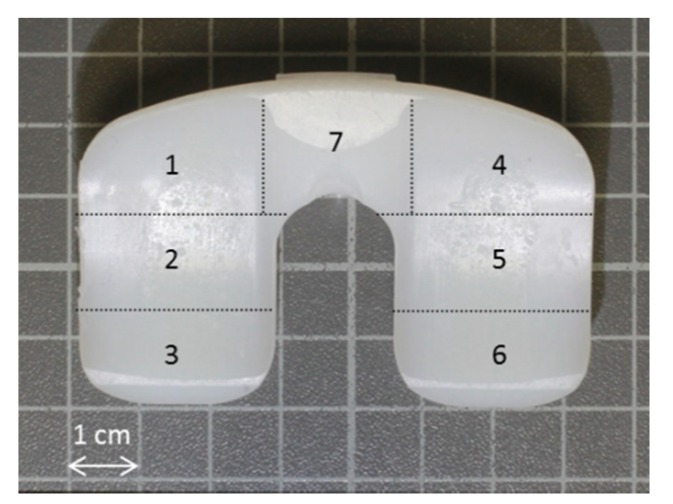
Polyethylene inlay with typical traces of wear. For the determination of the Hood score, the inlay was divided into seven zones.

Damage of polyethylene inlays was assessed on 40 inlays applying a modified Hood score [18]. We rated five damage mechanisms, i.e., delamination, pitting, scratching, burnishing and surface deformation at a scale between 0 (no damage) and 3 (distinct damage) for seven zones of the inlay, as shown in Figure 2. Achieved degradation scores were summed up, which results in a maximal possible score of 105. Determination of the modified Hood score was done by two investigators. The mean difference of the degradation score of the PE-inlays between the two investigators referred to 4.78 ± 3.2. The data presented refers to the evaluation of investigator 1.

### 2.3. Quantitative Wear Analysis by Coordinate Measurements

**Figure 3:**
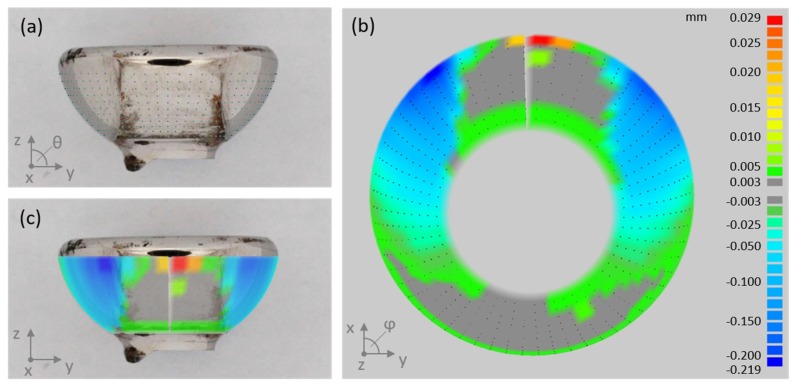
(**a**) Piston head (cut from the piston stem) with point cloud measured by CMM. (**b**) Color plot of linear wear, i.e., normal deviation between measured data and referencing surface. Gray areas refer to linear wear < 3 µm, which can be considered as unworn. (**c**) Overlay of linear wear plot and piston head image illustrates the unworn and most heavily abraded areas, respectively.

In order to estimate the material loss over the period of implantation, we assessed the geometry of the articulating surfaces of the coupling mechanism by a coordinate measuring machine (CMM; MS222, Mahr, Göttingen, Germany). For that, the sleeve and piston were separated by cutting the welding seam by which the lid was connected to the sleeve (see Figure 1b). Subsequently, the piston head was cut from the piston stem. The articulating faces of the semispherical piston head and the opposing counter face (Figure 1b) were scanned point wise in spherical coordinates with angle steps of 4° and 6° for theta (θ, polar angle) and phi (φ, azimuthal angle), respectively, as demonstrated for a cut off piston head in Figure 3a. Point clouds were further processed by Imageware^TM^ (UGS Corporation, Plano, TX, USA). As the original geometry of the measured parts is unknown, we did the analysis applying a best-case scenario. For this, we considered as much as possible of the spherical cap as un-worn and reasonable, and derived the referencing surface by fitting a sphere to these areas. Normal distances between each point and the fitted surface were derived (illustrated in Figure 3b,c) and further processed to determine the volumetric material loss. The latter was done using Matlab (Version 7.10.0, The MathWorks, Inc., Natick, MA, USA).

### 2.4. Statistical Analysis

Correlations of wear score and volumetric material loss, respectively, and time in vivo, bone resections length, patient weight and inlay degradation score, were assessed by Spearman’s rank order-correlation. The correlation of derived coupling wear scores from two independent investigators was assessed by the Pearson correlation coefficient. Differences in mean values ± standard deviation were tested for statistical significance by student’s t-test on unpaired samples with a level of significance of *p* ≤ 0.05. Statistical analyses were carried out applying SPSS software (IBM Corp., Version 22.0., Armonk, NY, USA).

## 3. Results

### 3.1. Failure Modes of Retrieved Implants

**Figure 4:**
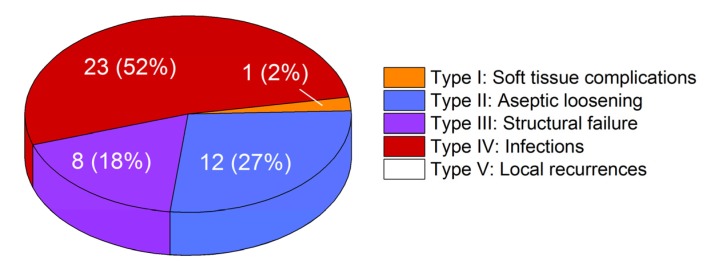
Distribution of failure modes according to the Henderson classification in our collective of 44 retrievals.

**Figure 5:**
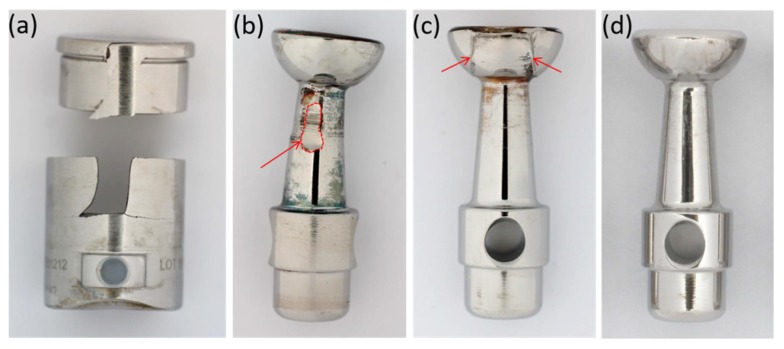
Damage occurring at couplings: (**a**) broken coupling sleeve, (**b**) bent piston with area of abrasive wear at piston stem (encirculated area, arrow), (**c**) piston head with zones of planar abrasion, the arrows mark the created edge between the worn and the unworn zones, (**d**) piston without noticeable planar abrasion.

Figure 4 represents the classification of the reasons for revision according to Henderson et al. [6]. 52% of the implants were revised because of infection (Henderson type IV). Aseptic loosening (Henderson type II) caused revision in 27% of the cases. Structural failure (Henderson type III), like breakage or joint instability due to worn couplings, occurred in 18% of the cases, while 2% were revised because of soft tissue complications (Henderson type I). No revisions were necessary because of local recurrences (Henderson type V). We found one case of piston breakage and four cases of sleeve breakage (see Figure 5a) in our collective. Three out of four explants with broken sleeves were revised for aseptic loosening. In one case, the piston was bent (see Figure 5b).

### 3.2. Evaluation of Wear by Damage Score

**Figure 6:**
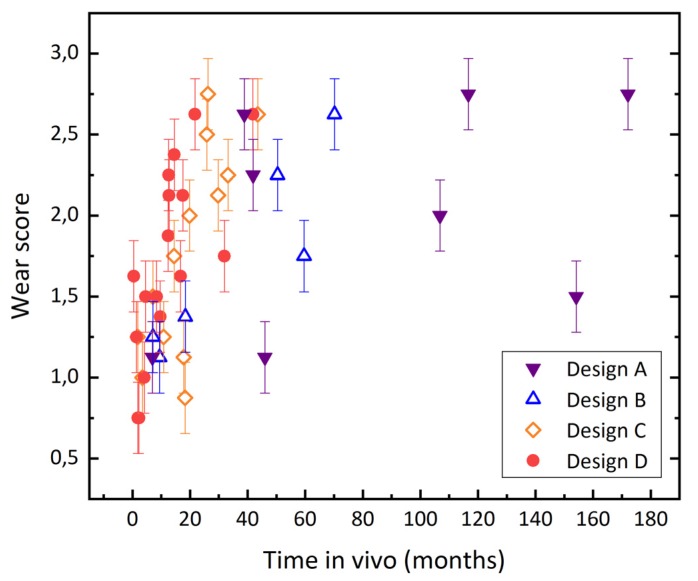
Coupling wear score increases with time in vivo.

Almost all retrieved MUTARS^®^ coupling mechanisms exhibit abrasive wear to some extent. The outside of the sleeves shows abrasion in the form of highly polished areas. Wear at the piston appears in the form of brush marks at the stem and head, as demonstrated in Figure 5. At the heads, abraded zones can be easily distinguished from unworn areas, as enough material is lost in the articulating zone to create an edge that separates the worn from the unworn zones at the originally semispherical surface (Figure 5c). According to the evaluation by damage score, 43% (n = 19) of the couplings were rated with a score of two or higher; i.e., they showed clear signs of planar abrasion. The mean wear score was 1.79 ± 0.62. The score increased with time in vivo (see Figure 6), with a Spearman’s rank-order correlation of r_s_ = 0.65, *p* < 0.01.

**Figure 7:**
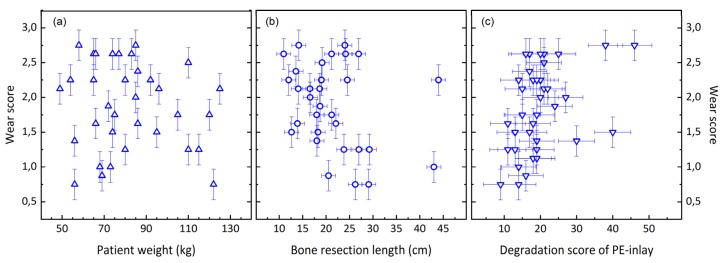
Scatter plots of wear score over (**a**) patient weight, (**b**) bone resection length and (**c**) degradation score of the polyethylene inlays.

We analyzed the correlation between the wear score of the coupling and patient weight, bone resection length and inlay degradation (scatter plots are displayed in Figure 7). Patient weight or bone resection length did not correlate with the damage score of the coupling (r_s_ = −0.02, *p* = 0.9 and r_s_ = −0.32, *p* = 0.06, respectively). The coupling wear showed a trend to increase with the degradation of the polyethylene inlay, as can be seen in Figure 7c; the Spearman’s rank-order correlation coefficient r_s_ amounted to 0.49 (*p* < 0.01).

Assessment of coupling wear by the two investigators showed a high correlation (Pearson r = 0.89). The mean difference in the coupling wear score of both investigators referred to 0.22 ± 0.19, while the median difference referred to 0.13, the minimum to 0, and the maximum to 0.75.

### 3.3. Quantification of Wear by Coordinate Measurements

**Figure 8:**
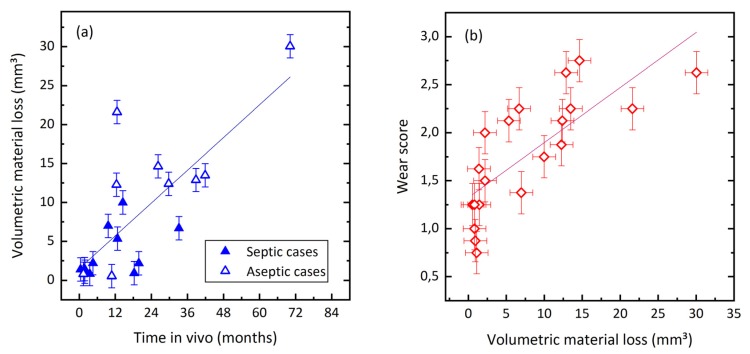
(**a**) Volumetric material loss increases with time in vivo. (**b**) Relationship between wear score and volumetric material loss.

Quantitative wear analysis of 20 retrievals estimated the volumetric material loss of the articulating surfaces of the coupling to range from 0.5 mm^3^ to 30 mm^3^. The total mean value accounted to 7.9 ± 8.1 mm^3^. The mean wear rate was 7.8 ± 8.6 mm^3^/year. Volumetric wear increased with time in vivo (Figure 8a), with a Spearman’s-rank order correlation of r_s_ = 0.69, *p* < 0.01. Wear score and volumetric material loss are correlated, as illustrated in Figure 8b (r_s_ = 0.867, *p* < 0.01).

Cases retrieved for joint infection showed 3.5 ± 3.2 mm^3^ of cumulated material loss, while the material loss of aseptic cases amounted to 13.2 ± 9.2 mm^3^. This can be attributed to the significantly shorter in-vivo periods of septic revisions, as shown in Table 2. Wear rates, i.e., volumetric material loss per time in vivo of both groups, are similar.

## 4. Discussion

The study investigated damage at a MoM rotating hinge mechanism due to abrasive wear with two different techniques: A semi-quantitative examination by which a coupling wear score is visually determined, and a CMM-based analysis that estimates actual volumetric wear. Quantitative measures on wear volume showed a relevant mean wear rate of 7.8 mm^3^/year. Both applied methods showed a clear relation between the extent of wear at the coupling and the time of in vivo service.

From Figure 6 it appears that the older designs (A and B) exhibited a better in vivo performance, because they show the longest periods of implantation, and the inclination of the wear score over in vivo service time seems to increase in the order of the design A, B, C and D. The shorter in vivo service time is related to the more recent implantations of the newer designs. Design D is implanted since 2015, design C was implanted between 2011 and 2014, design B between 2010 and 2012, and the retrievals with design A were implanted between 1997 and 2011. The available data, however, does not allow us to compare the long-term performance of designs B, C and D with the performance of design A, because there are no retrievals with coupling designs B, C or D that were implanted for more than 80 months in the investigated retrieval collective. Also, we do not know the number of well working implants that are currently implanted. A possible explanation for the difference in inclination could be that the highest wear rate occurs in the beginning of the in-vivo service. However, the quantitative analysis rather showed a linear relation, while the semi-quantitative correlation could also be interpreted as a logarithmic relation. We believe that this is mainly related to the fact that the semi-quantitative evaluation grades the extent of damage in finer steps in the beginning of the abrasive process. Brush marks at the piston head can be seen and were rated with a score of 1, while the actual volumetric material loss determined by the CMM evaluation can be still close to zero. As soon as the abrasive process led to the formation of the distinct edge between worn and unworn areas, the piston heads were rated with a score of 3, independent from the severity of the created edge.

The semi-quantitative analysis also rates those zones of the coupling that cannot be assessed by coordinate measurements, such as the outside of the sleeve. Here, abrasion is noticed visually in the form of areas with a highly polished appearance. Another advantage of the semi-quantitative wear analysis is its non-destructiveness. Coordinate measurements as quantitative alternative require dissociation of lid and bushing of the sleeve and of the piston head from stem.

It has been shown in simulator and retrieval studies, that wear and corrosion of metallic parts does occur, even in conventional, i.e., minimally stabilized, total knee arthroplasty. Considerable levels of metal ions are released during cyclic wear simulation [20]. Clinically, this leads to elevated metal ion levels in the patient’s blood [21,22,23]. In addition, the released metal ions accumulate in tissue surrounding the joint, and can lead to adverse local tissue reactions like metallosis [19,24]. This in turn can lead to aseptic loosening, which is always followed by a revision operation. However, quantitative data on metal wear from total knee arthroplasty is rare. Kretzer et al. report the total release of Co- Cr- and Mo-ions to be about 2.5 mg after 5 million cycles [20]. The wear rate of 7.8 mm^3^/year determined for the MoM couplings would correspond to a metal release into the human body of about 65 mg/year, which would be at least about 50 times the metal release from the articulating parts. Generally, clinical problems like adverse reactions to metallic debris (ARMD) or pseudotumor formation related to wear of CoCr-articulating surfaces are well known from MoM total hip arthroplasty (THA) [15,16,25]. Volumetric wear of retrieved MoM THAs has been examined in several studies. Lord et al. analyzed 22 retrieved cup-head MoM pairs, whereof the majority of patients had been revised for ARMD. The median of the determined wear rates was 9.4 mm^3^/year [25]. Glyn-Jones et al. found a significantly increased mean volumetric wear rate of 3.3 and 2.5 mm^3^/year for femoral heads and acetabular cups, respectively, in retrievals revised for pseudotumors compared to a no pseudotumor control group [26].

Grammatopoulos et al. confirmed these findings, which determined the total mean wear rate in the pseudotumor group to be 5.5 mm^3^/year, whereas the control group exhibited 0.4 mm^3^/year [16]. Gascoyne et al. reported a total median rate for metal loss from joint articulation and head-stem taper of 1.5 mm^3^/year in a retrieval study on 24 MoM hip replacements of a single design, with 16 of the 24 patients revised for MoM-related reasons, such as adverse reaction to metallic debris (ARMD) or high blood metal ion levels. The mean wear rate from articulating parts only referred to 4.6 mm^3^/year ranging from 0.01 to 66.4 mm^3^/year [27]. The mean amount of metallic wear of about 7.8 mm^3^/year created at MoM couplings in the MUTARS prosthesis is thus in the same order of magnitude than wear created from MoM hip TEPs that have been revised for metal-related causes. It is very likely that the debris created in the rotating-hinge system leads to similar metal-related problems, as it has been reported for failing MoM artificial hip joints. This hypothesis is supported by several studies that show considerably elevated metal ion levels related to hinged TKA: Laitinen et al. reported elevated Co- and Cr-ion levels of > 5 ppb in whole-blood samples of 19/22 patients with a MUTARS^®^ prosthesis applying a MoM coupling. In the control group with a MUTARS^®^ prosthesis using a metal-on-polyetheretherketone (PEEK) coupling, only 1 of 12 patients showed a similarly elevated Co-ion level [12]. Klasan et al. found > 5 ppb Co- and Cr-ions in the blood serum of 16/23 patients with a MoM hinge knee design [28]. Friesenbichler et al. showed elevated Co- and Cr-ion levels in serum blood for two hinged TKA systems (different from the MUTARS^®^-prosthesis), but not for a standard rotating hinge TKA [29].

A difficulty in the determination of material loss from retrievals is that the original geometry of the worn parts is unknown. Determining volumetric and linear wear therefore always requires estimation of the original geometry by a referencing surface or volume. The MUTARS^®^ rotating hinges are based on a ball joint, so the articulating faces can be represented by a spherical cap. We chose the referencing spherical face in such a way that as much of the measured articulation area as possible could be considered unworn, so we analyzed the best possible case for the coupling in terms of wear. In this way, resulting wear plots usually showed two unworn areas: The unworn, dorsally-oriented face not in contact with the bushing and the opposing ventrally-oriented face. As the latter actually is part of the joint articulation, it is very likely that it experienced abrasive wear to some extent, too. In addition, we determined the quantitative wear volume just from the articulating surfaces. Visually, we detected abrasion also on other parts of the hinge, such as the piston stem, the lid of the sleeve, the outside of the sleeve and on the rims of the openings in the sleeve. The calculated wear volumes therefore represent rather a minimum level of occurring metal abrasion.

In order to estimate how many revisions were caused by worn or broken bushings, we had a closer look on the reasons for revision: Seven prostheses out of 8 revised for ‘structural failure’ were explicitly revised for mechanical complications related to the coupling, such as instability due to bushing wear and material breakage. From the 12 cases that were revised for aseptic loosening, three cases showed broken coupling sleeves, and in four cases, metallosis was described in the operation report. Two of the metallosis-annotated cases coincided with the broken coupling sleeves. In the case of aseptic loosening with a diagnosed metallosis, we attributed implant loosening to the generated metallic wear. This would sum up to 12 out of 44 revisions (27%), which were probably related to the wear of the rotating hinge mechanism. In addition, five retrievals exhibited bushing or stem breakage, which results in a fracture rate of 11% in the investigated retrieval collective. About half of the investigated retrievals were revised due to an infection, which we did not consider to be related to metal wear. However, there are studies that suggest that metallic debris might be able to promote joint infections, as the tissue damaged by metallic wear products is an optimal environment for bacterial growth [30,31]. It should be noted that after a bone tumor resection at the proximal tibia or distal femur, which requires knee reconstruction by an endoprosthesis, musculature and soft tissue around the joint must be removed, and are therefore missing to stabilize the knee. The tumor endoprostheses systems are therefore subjected to much higher loads than the less stabilized TKA systems. The complications with the rotating hinge mechanism therefore have their reason at least partially in the underlying disease. However, an improved coupling design might be able to diminish occurring coupling wear and damage to some extent.

The study has several limitations. First of all, it is a retrieval study, meaning that we draw our conclusions only based on explants that clinically failed without any control group. Wear volume was estimated on the basis of a fitted referencing spherical surface, as the actual geometry of the measured parts is unknown. In addition, the spherical fit was done on rather small areas of the measured spherical caps. However, the total wear volume we calculated can be considered a minimum value, as we analyzed the measured parts under the estimation of a best-case scenario. Evaluation of coupling wear by damage score instead of quantitative analysis is subjective, and thus to some extent observer-dependent. Also, it does not scale linearly with the amount of abrasive wear.

## 5. Conclusions

This study investigated damage on the rotating hinge of a tumor and revision system (MUTARS^®^) for total knee replacements. Pronounced wear on the coupling mechanism occurs frequently, and was assessed by two different methods. Applying a scoring system has proven to be a reliable, non-destructive option to rate the degree of wear. It was shown that the extent of damage increases with time in vivo, but not with patient weight or bone resection length. Volumetric wear was estimated using coordinate measurements, and revealed high wear rates of about 8 mm^3^/year.

## Figures and Tables

**Table 1:** Criteria for the determination of the coupling wear score.

Score	Sleeve (Each Quadrant)	Piston Head	Piston Stem
0	No damage	No damage	No damage
1	Scratches and/or polishing < 10% of the surface	Scratches visible	Scratches visible
2	Polishing 10–50% of the surface	Planar abrasion noticeable	Planar abrasion < 10% of the surface
3	Polishing > 50% of the surface	Planar abrasion with distinct edge on the surface	Planar abrasion > 10% of the surface

**Table 2:** Total wear volume, time in vivo and wear rates of MoM couplings, subclassified into septic and aseptic revisions.

	Number of Cases	Time in Vivo (months)	Total Wear Volume (mm^3^)	Wear Rate (mm^3^/year)
	n	mean (SD/range)	mean (SD/range)	mean (SD/range)
Septic revision	11	10.9 (10.1/0.4–33.2)	3.5 (3.2/0.8–10.0)	8.2 (10.7/0.6–39.1)
Aseptic revision	9	34.3 (30.0/1.4–70.2)	13.2 (9.2/0.5–30.0)	6.7 (6.1/0.6–20.6)
*p*	−	<0.05	<0.05	0.7
All cases	20	19.4 (18/0.4–70.2)	7.9 (8.1/0.5–30.0)	7.8 (8.6/0.6–39.1)
